# The Recognition of Cross-Cultural Emotional Faces Is Affected by Intensity and Ethnicity in a Japanese Sample

**DOI:** 10.3390/bs11050059

**Published:** 2021-04-23

**Authors:** Andrea Bonassi, Tommaso Ghilardi, Giulio Gabrieli, Anna Truzzi, Hirokazu Doi, Jessica L. Borelli, Bruno Lepri, Kazuyuki Shinohara, Gianluca Esposito

**Affiliations:** 1 Department of Psychology and Cognitive Science, University of Trento, 38068 Rovereto, Italy; andrea.bonassi@unitn.it; 2 Mobile and Social Computing Lab, Fondazione Bruno Kessler, 38122 Trento, Italy; lepri@fbk.eu; 3 Donders Institute for Brain, Cognition and Behaviour, Radboud University Nijmegen, 6525 AJ Nijmegen, The Netherlands; t.ghilardi@donders.ru.nl; 4 Psychology Program, School of Social Sciences, Nanyang Technological University, Singapore 639818, Singapore; GIULIO001@e.ntu.edu.sg; 5 Trinity College Institute of Neuroscience, Trinity College, Dublin 2, Ireland; truzzia@tcd.ie; 6 Medical Engineering Department, Kokushikan University, Tokyo 154-8515, Japan; hdoi@kokushikan.ac.jp; 7 Department of Psychological Science, University of California, Irvine, CA 92697-7085, USA; jessica.borelli@uci.edu; 8 Department of Neurology and Behavior, Graduate School of Biomedical Sciences, Nagasaki University, Nagasaki 852-8523, Japan; kazuyuki@nagasaki-u.ac.jp; 9 Lee Kong Chian School of Medicine, Nanyang Technological University, Singapore 308222, Singapore

**Keywords:** face, face perception, emotion, facial affect, ethnicity, accuracy, recognition task, EEG, arousal, frontal asymmetry

## Abstract

Human faces convey a range of emotions and psychobiological signals that support social interactions. Multiple factors potentially mediate the facial expressions of emotions across cultures. To further determine the mechanisms underlying human emotion recognition in a complex and ecological environment, we hypothesized that both behavioral and neurophysiological measures would be influenced by stimuli ethnicity (Japanese, Caucasian) in the context of ambiguous emotional expressions (mid-happy, angry). We assessed the neurophysiological and behavioral responses of neurotypical Japanese adults (*N* = 27, 13 males) involved in a facial expression recognition task. Results uncover an interaction between universal and culturally-driven mechanisms. No differences in behavioral responses are found between male and female participants, male and female faces, and neutral Japanese versus Caucasian faces. However, Caucasian ambiguous emotional expressions which require more energy-consuming processing, as highlighted by neurophysiological results of the Arousal Index, were judged more accurately than Japanese ones. Additionally, a differential Frontal Asymmetry Index in neuronal activation, the signature of an approach versus avoidance response, is found in male participants according to the gender and emotional valence of the stimuli.

## 1. Introduction

Emotions are a source of critical information in our everyday life. Within meaningful social interactions, emotions are highly dense informative signals, conveyed by faces, and used to externalize implicit and explicit communicative intentions towards other individuals. From a cognitive-behavioral view, emotions are cognitive moderators which activate and regulate behavioral and physiological responses to social and environmental stimuli [1]. Emotions provide feedback on performance, filter important data from noisy sources, and provide global management over other cognitive capabilities and processes that are crucial in an ecological environment.

Charles Darwin [2] theorized the universality of facial expression, pointing out that emotions and their expression could be innate, and suggesting that similarities in facial expressions could be found phylogenetically. Darwin’s ideas were reinterpreted by Tomkins and McCarter [3], highlighting that emotions are the basis of human motivation and that this basis resides in the face. In 1964, they demonstrated that facial expressions could be associated with specific emotional states. This scientific evidence contributed to a first conceptual framing of the emotional expressions, defined as the set of behaviors and facial movements able to convey an emotional state. Carrying on Tomkins’ and McCarter’s studies, Ekman [4], Izard [5], and Friesen [6] laid the experimental foundations of the Universality Hypothesis conducting observational studies worldwide. A meta-analytic evaluation of emotional faces and other nonverbal stimuli disclose a universal ability to recognize emotions [7]. Interestingly, these studies conducted around the world between different laboratories, methodologies, and cultures seem to point to a unique and undeniable path: emotional expressions have evolved to be genetically rooted in us, maintaining similarities of structure and meaning across cultures and societies [8]. A body of expressions, which seem to represent the basis of our emotional ability, has been identified. Although other emotions were analyzed and discussed through different theoretical frameworks [9], the model of Ekman and Cordaro recognizes anger, contempt, disgust, fear, joy, sadness, and surprise as the seven main emotions [10]. Differently from the alternative models of basic emotions [11], this model conceives cultural learning as a predominant factor of emotional experiences. In line with the model, each of the mentioned basic emotions drives behavioral and physiological responses to respond to environmental requests appropriately and deal with ecological challenges [11].

The potential ubiquity in the recognition ability of emotional faces can also reflect the universality of emotional expressions. Common cognitive mechanisms and brain regions underpinning the emotional recognition of faces have been established in humans [12]. Although emotional face processing may be almost universal, the ability to express facial expressions can vary across ethnicities and may be influenced by the exposure to environment, culture, and society [13]. Numerous research demonstrated that the performance and the speed with which people are able to identify emotions seem to be influenced by the context and social categories, especially by ethnicity [14] and gender [15]. Ingroup advantages were proved by a variety of studies that demonstrated how individuals better recognize same-culture emotional expressions than emotional expressions from other-cultures [16]. An influential meta-analysis also revealed that there was an increase in accuracy when judging emotional expressions for members of a cultural ingroup [7]. For instance, Indian people exhibited a higher number of errors when classifying facial negative emotions like anger and fear compared to facial expression with positive meaning [17]. Again, happy expressions are most quickly and accurately categorized when those faces are female [18]. Faces are also rated as happier than average when the observer is exposed to a third-person happy expression during social interaction [19]. Overall, both affective and cognitive components of reported emotions may differ between people who belonged to Western and Eastern cultures [20]. Moreover, the interpretation of an emotion such as anger appears to depend on biases due to stereotypes towards targeted race and gender: European-Americans are faster and more prone to judge a face as angry when the target is a man or is black than when the target is a woman or white [21,22]. Nevertheless, the information from the environment might be more informative than the emotional valence conveyed by a given facial expression [23].

One of the factors mediating facial expression is the intensity of the conveyed emotion. Facial expressions are the result of multiple elements and are elicited for different reasons: from expressing the agony of losing a loved one to the frustration of having missed the last train. In everyday life, facial expressions of emotions are typically only of low to mid-intensity [24]. For this reason, ecological studies explored how the different modulations of emotional facial expressions can influence our recognition ability [25]. Extant studies have shown that even though the faculty of recognizing facial expressions is universal, emotions at distinct levels of intensity can be revealed differently between males and females and from culture to culture. In summary, even if facial expressions are globally shared and easily recognizable, several factors to a different extent lead to the ability to express emotions during social interactions in distinct parts of the globe.

Moreover, emotional facial expression can be processed on two different levels: explicit and implicit. In the majority of the cited papers up to this point, participants are presented with emotional facial stimuli and they are asked to choose the correct emotional label that, in their opinion, matches the facial expression. Thus, participants consciously perceive the facial expression and explicitly categorize it in one of the provided emotional labels. This task provides a straightforward measure of the observed behavior, based on the reported participants’ ratings. However, in everyday social interactions, facial expressions are recognized and interpreted without the explicit comparison with emotional labels, but they are implicitly processed [26]. The distinction between implicit and explicit processes is considerable not only to classify two methodological approaches, but also given different neural activations that these two processes seem to elicit [27]. Differently from emotional recognition, which refers to the explicit ability through which a given emotion is identified at a certain level of accuracy by the perceiver, emotional elicitation is the implicit condition resulting from observing a facial expression that evokes a given emotional feel and arousal.

The implicit process, combining the multiple dimensions that define facial expressions and the contexts they are expressed in, takes place in the central nervous system. Numerous studies have analyzed the central nervous response to emotional facial expressions from EEG rhythmic activity [28]. Most emotion responses were found in the Alpha band with the right hemisphere reflecting responses to negative emotions, such as fear or disgust, and the left hemisphere reflecting reactions to positive emotions, such as happiness [29]. Palmiero and Piccardi [30] explained that the valence of the emotion is associated with asymmetry in the frontal lobe and that the arousal is associated with a general activation of both the right and left frontal lobes. Additionally, significant differences in the EEG power and hemispheric asymmetries can be observed in processing distinct emotions [31].

To better understand the multi-factorial interaction that influences the expressions we are exposed to daily, we aimed to explore the relationship between explicit and implicit processing underlying the recognition of clear versus ambiguous facial emotion expressions in own versus other ethnic groups. To this end, we assessed the central nervous system and behavioral responses of Japanese adults to facial expressions that varied in gender, ethnic group, emotional valence, and intensity.

Given the role of culture in influencing the implicit processing of emotions and the use of mild facial expressions—which should require a higher attentional demand—we expected that different accuracy scores and neurophysiological activation would be observed in response to Japanese versus Caucasian stimuli. Specifically, previous findings [32] suggested that accuracy in the emotional recognition was higher in people who judged faces of the same ethnic group. Considering that this ingroup advantage was probably due to a cultural exposure effect, we hypothesized that accuracy scores of Japanese people would be higher in response to ambiguous Japanese emotional faces than Caucasian ones. In contrast, regardless of the expressed emotional valence, we expected that our sample would show a dissociation between accuracy scores and neurophysiological activation in the prefrontal cortex to faces.

Previous researches found cross-cultural differences in emotion-related physiological arousal. In the collectivist societies typical of Eastern culture, people tend to conform to others and generally maintain low levels of arousal independent of the emotional context [33]. However, further research is required to understand whether Eastern individuals’ arousal patterns (e.g., Japanese people) vary to cross-cultural emotional stimuli at different levels of ambiguity. Regarding the current study, we expected an increase in the ratio between Beta and Alpha waves (a neurophysiological response here called “Arousal index”) to Caucasian ambiguous faces compared to clear ones. More specifically, in the context of ambiguous emotional expressions, here we hypothesized that arousal as a biomarker of enhanced neurophysiological distress in the prefrontal cortex would be higher to equivocal Caucasian faces than clear ones independent of their emotional valence.

Another research line has focused on the hemispheric asymmetries of the prefrontal cortex in emotional processing [30]. According to the motivational direction hypothesis, a high Alpha band activity in the left prefrontal cortex has been associated with the motivation to approach the emotional stimulus (e.g., face). Vice versa, a high Alpha band activity in the right prefrontal cortex has been linked to the motivation to withdraw from the emotional stimulus [29]. With regards to the present research, we expected that the ratio between right Alpha and left Alpha waves (a neurophysiological response here called “Frontal Asymmetry Index”) would decrease to Caucasian ambiguous faces compared to Japanese ones. In particular, here we hypothesized that a low frontal asymmetry would explain a withdrawal disposition of Japanese people from Caucasian faces, as they are perceived as more adverse and hostile than ones belonging to the same ethnic group.

### Significance

The proposed research paradigm opens to four innovative aspects, which are remarkable in providing further contributions to the discipline. The current study: (a) investigates the multiple factors which simultaneously affect the ability to express emotions; (b) measures both behavioral and neurophysiological responses to emotional faces in identifying potential similarities—or differences—among implicit and explicit measures; (c) evaluates the influence that the ethnic group may have in decoding an ambiguous emotional intensity facial expression [33], undermining the Universality Hypothesis [34]. Generally, people are continuously exposed to the same ethnic group’s faces from an early age and across their entire lifespan. However, the exposure to faces of a different ethnic group—which represent less common stimuli—reveals inferior expertise in facial expressions’ emotional discrimination, especially if equivocal.

## 2. Methods and Materials

### 2.1. Participants

Japanese adults (*N* = 27, 13 males) aged 19–30 voluntarily enrolled in our study (mean age = 23.48, *SD* = 3.89). Exclusion criteria were: current or lifetime history of neurological or psychiatric disorder, dominant and preferential use of the left hand, and age higher than 30 years. Limited to just the sample size of EEG data processing, as we found technical problems in the recorded signal of 1 participant, later discarded, there is one fewer participant data analyzed (*N* = 26, 12 males, mean age = 23.62, *SD* = 3.90) . The research was approved by the Ethical Committee of Nagasaki University (IRB number 05053035-13). Informed consent was obtained by all the participants.

### 2.2. Stimuli

**Figure 1:**
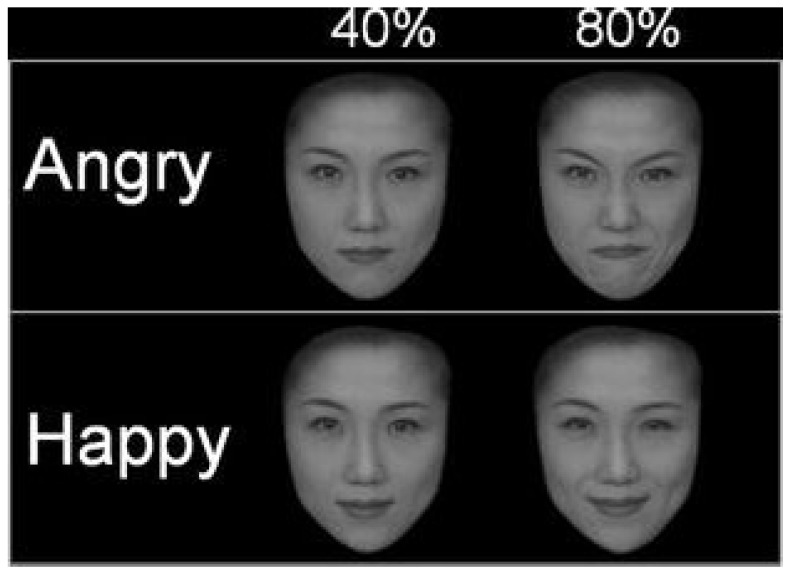
From Doi et al. [36]. Example of the 2 emotions expressed with the low and high intensities by a Japanese actress (40% and 80% of emotional intensity, respectively). Permission to use this figure was obtained from the author(s) of the publication [36].

Four emotional facial pictures of Japanese male and female adults and four emotional facial pictures of Caucasian male and female adults were respectively obtained from the standardized emotional expression databases ATR DB99 (ATR-Promotions) and Karolinska Directed Emotional Faces [35]. Applying the commercial morphing software FUTON (ATR-Promotions), emotional facial expressions were manipulated at increasing levels of intensity. FUTON provides intermediate facial expressions by merging two extreme expressions taken from the same individual with pre-defined proportions. Facial expressions with lower emotional intensity can be realized by decreasing the proportion of the influence of a given emotional expression, and vice versa. Here, graded facial expressions with two levels of emotional intensity were created by merging neutral (0%) and full angry and happy facial expressions (100%). The proportions of the influence of each emotional expression were set at 0% (original picture with neutral facial expression), 40% and 80% (Figure 1).

Overall, faces varied according to gender (male versus female), ethnicity (2 ethnic groups: Japanese versus Caucasian), emotional valence (2 emotions: happiness versus anger), and intensity (3 levels: 0% versus 40% versus 80%) for a total of 48 stimuli (4 actors × 2 ethnic groups × 2 emotions × 3 intensities). Angry and happy faces were adopted because of their hard-wired evolutionary meaning and their property of eliciting physiological neurophysiological responses. The low (40%) and high (80%) intensities were picked to reproduce an ecological situation for which the recognition of facial expressions would have been demanding [36], while the 0% intensity (lack of emotional valence) was preserved as a control variable and baseline for the calculated behavioral and neurophysiological indexes. Conversely, expressions at 100% intensity were excluded as univocal to recognize and scarcely informative for this ecological experimental design. Indeed, in everyday life, human ability to manifest emotions plays an essential role especially when emotional expressions are mitigated by environmental factors and could be perceived as ambiguous.

In the context of an ecological research paradigm, motionless pictures of emotional facial expressions were preferred rather than video clips of emotional faces for several reasons: (a) the pictures used in the current study were previously validated [37] and achieved a broad recognition by the scientific community; (b) the adoption of stationary faces allowed us to maximize the control of the intensity manipulation for each emotional expression [36]; (c) fixed emotional faces are guaranteed to keep high standardization of facial features across conditions.

### 2.3. EEG

EEG signals were recorded using a 64 electrode setup sites referenced to the vertex using a HydroCel Geodesics system (Electrical Geodesics, Inc., Eugene, OR, USA). The EEG signal was sampled at 1000 Hz and band-pass filtered between 0.1 Hz and 200 Hz. The signal was stored on an external device (iMac G5) for subsequent analysis. The system consists of an array of sensors arranged in an elastic tension structure, which can be relatively quickly and easily slipped on and off the participant’s head.

### 2.4. Facial Expression Recognition Task

**Figure 2:**
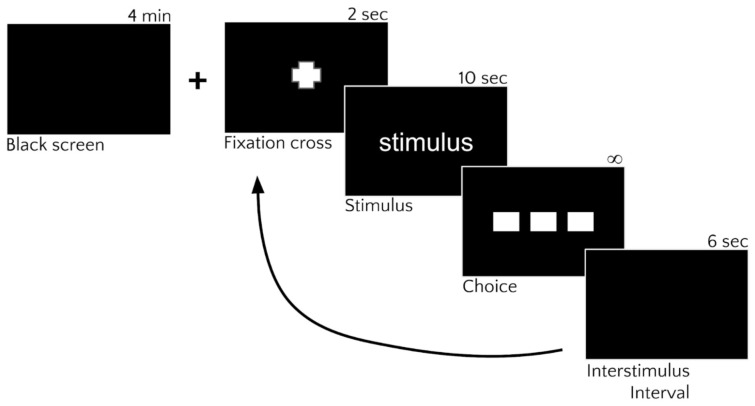
Graphical representation of the experimental design. After four-minutes of baseline recording, four blocks (each formed by 12 epochs) were presented to each participant. Every epoch consisted of the following steps (from top to bottom): (1) A centered two-second fixation cross prepared the participant for the stimulus. (2) A 10-second stimulus appeared in the center of the black screen. (3) Three buttons labeled with kanji (happiness, anger, and neutral) were presented on the screen. The participant was asked to specify the facial expression’s emotional category as accurately as possible, with no time constraint, by pressing with the mouse on the correct kanji label. (4) A six-second black background of the inter-stimulus interval was displayed.

Figure 2 represents the experimental design graphically. At the beginning of the experiment, 48 faces were randomly divided into 4 blocks (Matlab, The MathWorks Inc., Natick, MA, USA). After 4 min of baseline recording, the 4 blocks (each formed by 12 epochs) were presented to each participant. For each epoch, a centered 2-s fixation cross preempted the participant to the appearance of the stimulus. After the cross, a 10-s stimulus appeared in the center of the screen against a black background. After the stimulus’ presentation, 3 buttons labeled using kanji were presented on the computer screen. Here, participants were instructed to specify the emotional category of the facial expression by pressing with the mouse on the correct key among the three different kanji labels (happiness, anger, and neutral). The position of the labels on the monitor was randomly assigned at the beginning of the experiment and was stable across blocks. Participants were asked to provide their responses as accurately as possible with no time constraint. After their response, a 6-s black background of inter-stimulus interval was displayed on the monitor.

Once the interval has ended, the cycle is repeated starting with another fixation cross. Each block lasted around 4 min (around 20 s × 12 epochs) and the total experimental session was conducted in 50 min.

To achieve a multi-dimensional insight of emotional processing, we recorded both the behavioral response as an explicit measure of the emotional face recognition task and the electroencephalographic signal (EEG) as the implicit measure.

## 3. Methods and Techniques

Analyses have been performed to explore both explicit and implicit indexes in order to identify any influence of the independent variables and their interaction.

For each measure, the processing of raw data was conducted using Python (version: 3.6.4), whereas the statistical computation was run on R (version: 3.5.1).

### 3.1. Behavioral Response: Processing and Statistics

To study participants’ behavior in response to the presented facial stimuli, an index of accuracy was permuted for each variable of interest. The behavioral accuracy was chosen as a simple and reliable descriptive metric for multi-class classification (three response choices among happiness, anger, and neutral were possible) rather than alternative measures, which would have been more sensitive to extreme values (e.g., flattening results into high percentages) (Future research could compute additional prediction metrics, e.g., accuracy, precision, recall and provide further behavioral measures, e.g., response time). For every participant, each generated variable was created by calculating the number of correctly recognized stimuli within a specific condition, divided by the total number of stimuli which belonged to the same condition. The formula of accuracy can be formalized as follows:

NumberofCorrectlyRecognizedstimuliinthecondition/TotalStimuliofthecondition

Therefore, a variable was described by a list which included a percentage value of correct recognition for each participant. To understand which type of analysis could be used, we visually checked power values for their distribution (the R library “e1071” was implemented). Most of the permuted dependent variables of accuracy displayed a normal distribution, except for the accuracy to Caucasian neutral faces (skewness = −2.43), the accuracy to high intensity emotional faces (skewness = −2.80), and the accuracy to Japanese low intensity angry faces (skewness = 1.31). We applied log transformation to those variables which were not Gaussian distributed. Although the distributions were improved by the log correction, the original distributions were preserved in order to maintain proper data interpretation and clear informative data representation of the behavioral variables prior to the data analysis. Consequently, we further checked that Student’s *t*-tests on log-transformed variables showed the same effects to be sure that no significant statistical differences could be attributable to the nature of the distribution.

In order to test six behavioral hypotheses, we first statistically compared the main conditions for each considered variable. Here, one unpaired and five paired Student’s *t*-tests were run to verify differences in the accuracy between groups. Additionally, in order to test our hypotheses, we also statistically compared the conditions within the interaction between our target variables: ethnicity, emotion, and intensity. Here, six paired Student’s *t*-tests were run to check for differences in the accuracy between groups. To take into account the multitude of tests performed, a Bonferroni correction was applied (12 repeated measures, α=0.0042) (Future studies could adopt more robust approaches such as the permutation tests in the context of pairwise comparisons [38]. As an alternative to multiple tests, β regression modeling would be optimal given accuracy and error rates bounded between 0 and 1).

Moreover, we applied the Stepwise Model Path function (stepAIC) [39] to a first logistic regression with accuracy as the dependent variable—defined by logic values (True versus False)—and emotion, intensity, and ethnicity as independent factorial variables (the R libraries “MASS” and “pscl” were used). From the initial logistic regression in input, the stepAIC reproduces an Analysis of Deviance Table and, for a specific dependent variable, it derives the most appropriate independent variables in output. In our analysis, stepAIC printed intensity and ethnicity as the more suited independent variables to maximize the prediction on the behavioral response accuracy. Therefore, we ran a final logistic regression with accuracy as the dependent variable and intensity and ethnicity as independent variables in interaction.

Finally, Cohen’s *d* and odds ratio were calculated to assess the size of the significant effects for *t*-tests (the R library “compute.es” was adopted for the effect size calculation of the only unpaired Student’s *t*-test) and logistic regression, respectively.

### 3.2. EEG Processing

The EEG data were analyzed offline using “MNE” (version: 0.15) [40,41], an Open-source Python package designed for the analysis of EEG signals.

Signals were band-pass filtered (0.2–45 Hz) to remove external sources of noise, and subsequently downsampled to 250 Hz. Continuous data were segmented in epochs of sixteen seconds (6 s pre-stimulus + 10 s stimulus). Epochs containing bad signal were automatically detected, then repaired or discarded using “autoreject” [42,43]. Following artifact rejection, data were re-referenced to the average of all channels and visually inspected to assess the presence of undetected bad channels.

Using the Multitaper method, with a variable number of cycles, the power values of Alpha (8–12 Hz) and Beta (12.5–30 Hz) [44] were extracted using a Fast Fourier Transform (FFT) and baseline-corrected. Specifically, the 10-s stimulus-related power was divided by the 5-s pre-stimulus power for both frequency bands (last 3 s of the inter-trial interval + 2 s fixation cross).

For each epoch, a single power value was calculated by integrating the power over the 10-s post-stimulus. Compared to the sum of the power, which is calculated by a limited number of time points corresponding to a specific factor of the FFT, the integer of the power considers the evolution of the signal over time and space between consecutive points of the signal curve. For instance, the integer of the power provides more precise and specific insight into temporal and hormonal dynamics because it considers the dynamics of the signal curve both in its increasing and decreasing phases. This time window was chosen in order to maximize the averaged power of the frequency bands signal in the whole duration of each stimulus presentation.

### 3.3. Arousal Index

**Figure 3:**
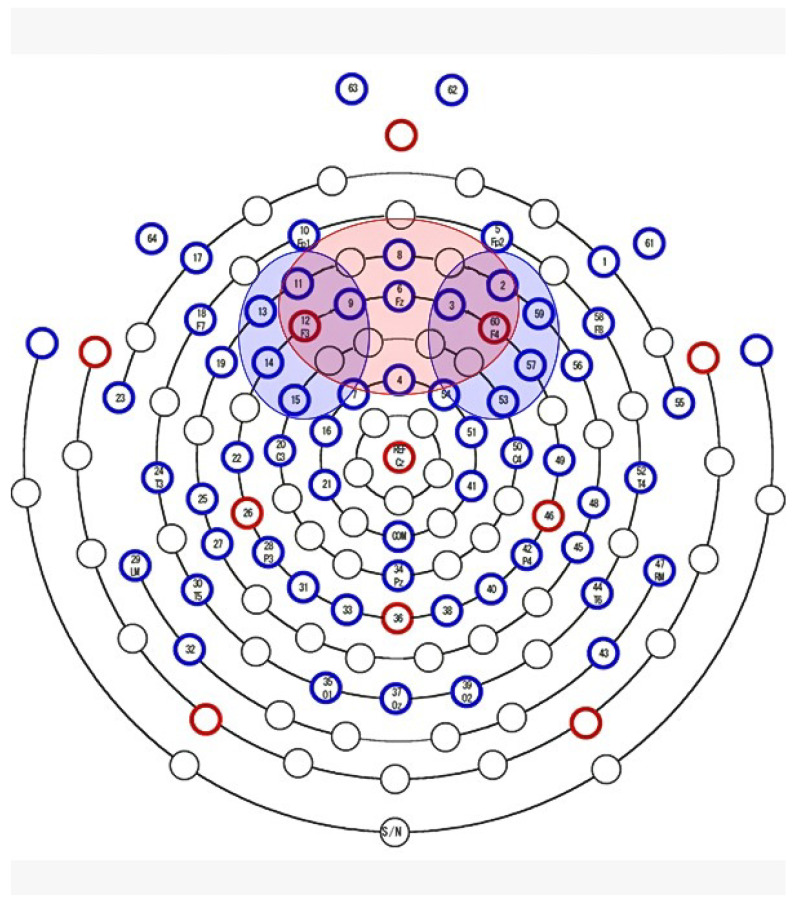
Location of each electrode on the brain scalp. The red area defines the central cluster (E2, E3, E4, E6, E8, E9, E11, E12, E60) considered for the Arousal Index. Regarding the Frontal Asymmetry Index, the left blue area delimits the left hemisphere’s cluster (E9, E11, E12, E13, E14, E15), and the right blue area depicts the right hemisphere’s cluster (E2, E3, E53, E57, E59, E60).

Beta waves are generally associated with an active state of mind and represent the state of alertness and wakefulness, while Alpha waves are more dominant in relaxing and pleasant contexts [45]. Alpha and Beta waves are linked by an inverse relation during cortical activity where an increase in Alpha activity brings about a decrease in Beta activity, and vice versa [46]. Emotions can be elicited in the brain when the individual is exposed to an emotional stimulus. Earlier contributions reported that humans show an increase in Beta responses during the emotional processing [47]. The ratio between Beta and Alpha computed on a central cluster of the prefrontal cortex (we considered E2, E3, E4, E6, E8, E9, E11, E12, E60; see channels distributions in the red area of Figure 3) could be seen as a parameter to monitor the state of arousal and of activation the subject is in [48] and it is described by the formula:

$$\ln(\frac{prefrontal\;cortex\;betapower}{prefrontal\;cortex\;alphapower})$$

We called this index “Arousal” since it has been used in emotional elicitation paradigms [49]. This index interestingly considers the simultaneous variation of Beta and Alpha bands to measure the neurophysiological involvement of the participant with emotional stimuli. An increase in this index suggests that the neurophysiological arousal could be related to the presented emotional faces.

### 3.4. Frontal Asymmetry Index

The Frontal Asymmetry Index is an index that describes the tendencies to approach or withdraw from a presented stimulus [29]. This index is computed by analyzing the activation of the left prefrontal cortex, associated with approach tendencies and appetitive emotions, and the right prefrontal cortex, which is linked to withdrawal tendencies and aversive emotions. According to the affective models, decreased left frontal activity also indicated greater mere exposure effect, suggesting that familiar stimuli are rated as more likeable and more associated with positive outcomes and approaching propensities [50]. Frontal asymmetry is commonly extracted from Alpha power values of F4 and F3 electrodes that respectively represent the right-prefrontal and left-prefrontal lobe; nevertheless, the use of clusters around those main electrodes is widely accepted and employed [51]. Frontal Asymmetry Index was then extracted using the formula:

$$\ln(\frac{right\;prefrontal\;cortex\;alpha\;power}{left\;prefrontal\;cortex\;alpha\;power})$$

Due to its properties, Frontal Asymmetry Index has been extensively used in emotional protocols as an indicator of the implicit value that the subject assigns to the stimuli [52]. Most of the studies have focused on how this index correlates with emotional and motivational processing associated to human faces [53]. In our study, Alpha power values of six close prefrontal cortex electrodes were mediated for each hemisphere in order to obtain an extended representation of the electrical activity of the area. A left hemisphere’s cluster (E9, E11, E12, E13, E14, E15; see channels distributions in the left blue area of Figure 3) and a right hemisphere’s cluster (E2, E3, E53, E57, E59, E60; see channels distributions in the right blue area of Figure 3) were generated. Datapoint differing more than two standard deviations from the mean were identified as outliers and removed from subsequent analysis.

### 3.5. EEG Statistics

For each neurophysiological measure, the average levels of frontal asymmetry and arousal were calculated for each stimulus presentation. Prior to data analysis, the distributions of frontal asymmetry and arousal values were inspected for normality.

Two repeated measure ANOVAs were conducted on the clustered Frontal Asymmetry Index and Arousal Index to angry and happy stimuli using emotion, intensity, ethnicity, and sex of the actor as within-subject variables and the participant sex as a between-subjects variable, while two repeated measure ANOVAs were conducted on clustered Frontal Asymmetry Index and Arousal Index to neutral stimuli using ethnicity and sex of the actor as within-subject variables and the participant sex as a between-subject variable. α was fixed at 0.025 for the Frontal Asymmetry Index and Arousal Index. Therefore, for the neurophysiological dependent variable “Arousal”, one 2-way interaction effect was evaluated, while for the neurophysiological dependent variable “Frontal Asymmetry”, one 3-way interaction effect was considered. With regards to the interaction effect on arousal, a one-tailed post-hoc Welch’s *t*-test (α=0.05) was run, while for the interaction effect on Alpha frontal asymmetry, one post-hoc paired Student’s *t*-test and one Welch’s *t*-test (corrected α=0.025) were carried out within groups. Partial eta squared (the R library “sjstats” was implemented) and Cohen’s *d* were calculated to estimate the size of the significant effects for ANOVAs and the post-hoc tests, respectively.

## 4. Results and Analysis

### 4.1. *Behavioral Results*

#### 4.1.1. Main Effects: Student’s *t*-Tests

Means and standard deviations of the measured variables are reported in Table 1.

**Figure 4:**
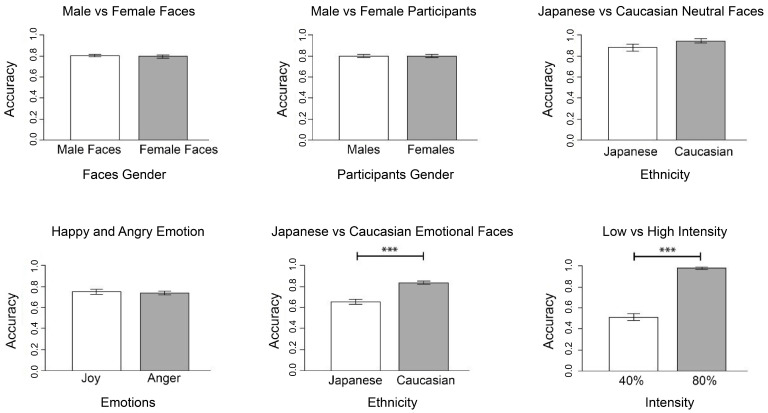
Comparison between main conditions’ accuracy rate: male and female faces, male and female participants, Japanese and Caucasian neutral faces, happy and angry emotion, Japanese and Caucasian emotional faces, low and high intensity. (*** *p* < 0.0042).

Student’s *t*-tests were performed to verify the presence of significant differences in the accuracy between groups (Figure 4).

As expected, no significant difference in the accuracy scores was found between male versus female participants (*t* = −0.05, df = 25, *p* = 0.96, *d* = −0.02), male versus female faces (*t* = 0.66, df = 26, *p* = 0.51, *d* = 0.16), neutral Japanese versus Caucasian faces (*t* = −1.71, df = 26, *p* = 0.1, *d* = −0.44), and happy versus angry faces (*t* = −0.44, df = 26, *p* = 0.66, *d* = −0.10). However, a significant difference was found in participants’ behavioral response to Japanese versus Caucasian emotional faces (*t* = −7.61, df = 26, *p* < 0.001, *d* = −1.70). Although we did not expect it, accuracy scores were higher in response to Caucasian emotional faces. Additionally, a significant difference was found in accuracy scores in response to different emotional intensities (*t* = 14.76, df = 26, *p* < 0.001, *d* = 3.87), indicating that more definite emotional faces were better recognized than more ambiguous ones.

#### 4.1.2. Interaction Effects: Student’s *t*-Tests

**Figure 5:**
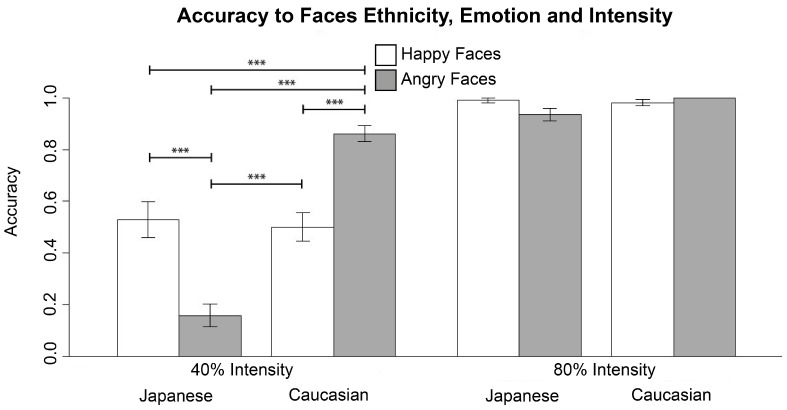
Contrast between accuracy rates to faces at 40% of intensity: Japanese happy and angry faces, Caucasian happy and angry faces. Accuracy rates to faces at 80% of intensity are represented: Japanese happy and angry faces, Caucasian happy and angry faces. No comparisons between conditions with faces at 80% of intensity were performed. Bars of happy faces are white-colored, while bars of angry faces are gray-colored. (*** *p* < 0.0042).

Stimuli with clearer emotional expression (80% intensity) were recognized with high accuracy levels independent of ethnicity and emotional valence. Taking into account only more ambiguous stimuli (40% intensity), both an effect of ethnic group and of the expressed emotion were instead found (Figure 5).

Contrary to our expectations, accuracy scores in response to Japanese angry faces were lower than scores in any other condition (Japanese-Angry versus Japanese-Happy: *t* = 5.12, df = 26, *p* < 0.001, *d* = 1.22; Japanese-Angry versus Caucasian-Happy: *t* = 5.12, df = 26, *p* < 0.001, *d* = 1.32; Japanese-Angry versus Caucasian-Angry: *t* = −13.60, df = 26, *p* < 0.001, *d* = −3.54). Furthermore, accuracy scores in response to Caucasian angry faces were also higher than accuracy scores in response to happy faces, independent of the ethnic group (Caucasian-Angry versus Caucasian-Happy: *t* = −5.60, df = 26, *p* < 0.001, *d* = −1.56; Caucasian-Angry versus Japanese-Happy: *t* = −4.56, df = 26, *p* < 0.001, *d* = −1.19). No significant difference in the accuracy score was found between Japanese happy versus Caucasian happy faces (*t* = 0.36, df = 26, *p* = 0.73, *d* = 0.09).

#### 4.1.3. Logistic Regression

Significant main effects of intercept (β=0.76, SE = 0.15, *z* = 5.18, *p* < 0.001, OR = 2.13), ethnicity (β=−1.41, Std. Error = 0.21, *z* = −6.88, *p* < 0.001, OR = 0.25) and intensity (β=3.92, Std. Error = 0.73, *z* = 5.40, *p* < 0.001, OR = 50.22) were found, while the interaction between ethnicity and intensity was not significant (β=−0.01, Std. Error = 0.82, *z* = −0.01, *p* = 0.99, OR = 0.99).

### 4.2. *Neurophysiological Results*

**Figure 6:**
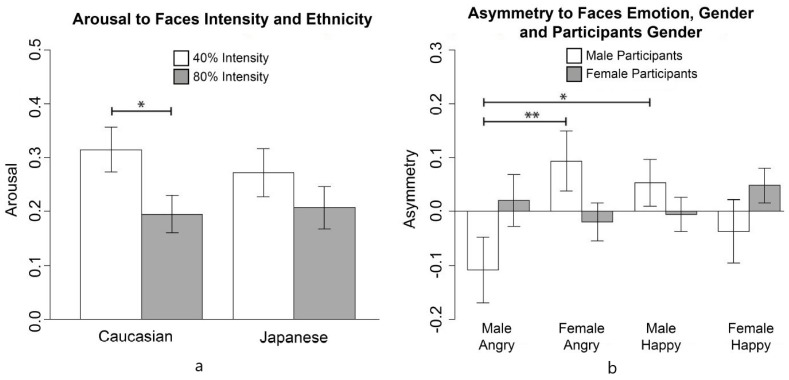
(**a**) Comparison between arousal in 40% and 80% intensity divided in Caucasian and Japanese (**left** figure). (**b**) Comparison between asymmetry in male and female participants divided according to stimulus’ emotional valence and gender (**right** figure). (* *p* < 0.02, ** *p* < 0.01).

Taking into account only stimuli with emotional expressions, a significant two-way interaction between intensity and ethnicity emerged for Arousal Index (*F*(1,25) = 5.86, *p* < 0.02, p$\eta^{2}$ = 0.02) (see Figure 6).

In line with our hypothesis, the arousal was higher in response to the Caucasian ambiguous stimuli than the univocal ones of the same group (*t* = 2.21, df = 196.68, *p* = 0.01, *d* = 0.31). Moreover, a significant three-way interaction between emotion, faces’ gender, and participants’ gender emerged for Frontal Asymmetry Index (*F*(1,25) = 10.35, *p* = 0.001, p$\eta^{2}$ = 0.03) to emotional stimuli. Specifically, male participants showed negative asymmetry in response to male angry faces which was significantly different from the positive asymmetry they showed in response to female angry faces (*t* = −2.49, df = 45, *p* < 0.01, *d* = −0.51) and male happy faces (*t* = −2.16, df = 82.17, *p* < 0.02, *d* = −0.45).

In contrast with our predictions, no effect of ethnicity and intensity was found on Frontal Asymmetry Indexes.

## 5. Discussion

In the present study, we explored the multifactorial nature of facial expression recognition and perception by investigating Japanese males and females behavioral (accuracy levels) and neurophysiological responses (arousal, frontal asymmetry) to ambiguous facial expressions that varied in gender, ethnic group, and emotional valence. We hypothesized that participants would be more accurate in correctly recognizing the valence (angry, happy, or neutral) of ambiguous emotional faces (40% happy, 40% anger) of the same ethnic group (Japanese) than the opposite group (Caucasian). Participants were also expected to show a higher neurophysiological Arousal and a lower Frontal Asymmetry Index to Caucasian ambiguous faces compared to Japanese ones independent of their emotional valence.

Japanese male and female participants did not significantly differ in their ability to properly recognize the emotions expressed. We also found no main effects of gender or emotional valence on explicit judgments. A general lower accuracy in response to ambiguous versus univocal emotional faces was found and served as a manipulation check, underlying a higher complexity in the emotional decoding of a face expressing an ambiguous or partial emotion independently of its valence. Specifically, both multiple *t*-tests and logistic regression detected the main effects of the ethnic group and of the emotional intensity on accuracy levels. Significant mean differences of the performance between conditions revealed that the same interaction between the ethnic group and the emotional intensity modulates the behavioral performance. In particular, this effect corroborates our starting hypothesis and seems to be driven by participants’ responses to ambiguous angry expressions. The current results broaden previous findings [54], highlighting that East Asian observers seem to bias the categorization responses towards Caucasian facial expressions [55], especially negative ones, and showing that this bias could only emerge when perceiving ambiguous stimuli [56]. Interestingly, this effect seems to differ between the two ethnicities: ambiguous angry faces expressed by Caucasian faces were recognized with the highest accuracy, whereas they were the most poorly judged stimuli when expressed by Japanese faces.

In accordance with the hypotheses, Caucasian ambiguous faces also elicited a stronger arousal response compared to univocal Caucasian faces. Indeed, faces with perceptually-ambiguous emotional expressions, independent from their valence, prompt a higher increase in the arousal response [57]. This result supports our hypothesis according to which low-intensity Caucasian faces could disclose high cognitive and behavioral engagement in processing multiple complex and equivocal features in a daily ecological environment [58,59]. Caucasian members’ ambiguous facial expressions could induce more than an automatic and spontaneous cognitive process: the higher arousal is a neurophysiological stress signature which could be associated with a higher cognitive loading in disclosing the significant emotional features associated to the facial expressions. This statement is consistent with researches which indicated that the perceived level of arousal modulates multiple physiological and neurophysiological responses to faces, independently of their emotional valence [60].

On the other hand, the valence and gender of the stimuli, but not their intensity or ethnic group as expected, differently affected the asymmetrical activation of the frontal lobes in males versus females participants [61]. Male participants showed a negative Frontal Asymmetry Index in response to male angry faces that significantly differed from the positive Frontal Asymmetry Index in response to male happy faces and female angry faces. Therefore, males may show an avoidant response towards male angry faces and an approaching response towards happy ones of the same sex and angry ones of the opposite sex [29].

### 5.1. Limitations

Several limitations may be evident from the present work. Firstly, the sample size was small as a consequence of the time constraints of the data collection. Secondly, the current research focuses on Japanese participants’ recognition ability towards Japanese and Caucasian faces. Since the sample did not include individuals from the Caucasian ethnicity, cross-cultural analyses were not performed between ingroup and outgroup, making the results more difficult to be generalized. Thirdly, although the faces with absent (neutral) and full emotional expressions (100% happy and angry) were obtained from two highly recognized standardized databases of actors’ expressions, graded facial stimuli (40% happy and angry, 80% happy and angry) were artificially handled by a morphing software. This manipulation was also applied from previous studies [36] and represented a simulated approximation of a real-life emotional expression that could affect the paradigm with potential biases. A fourth limitation emerged from the restricted number of emotions that were selected a priori. Happiness and anger, associated with distinct cognitive and neural pathways, are the most considered in affective neuroscience research. However, the probability of detecting the correct emotion in a facial recognition task could decrease up to 50% when only two response choices of emotions (happiness, anger) plus neutral are available. Moreover, the neutral response could also be selected from participants who were doubtful or prefer to keep a more conservative choice rather than guess the visualized facial expression’s emotion. Presenting facial expressions of two similar emotions (e.g., fear and anger) and controlling the related response choices could lead to more consistent results. Therefore, testing a more extended set of emotions (i.e., at least three among sadness, contempt, disgust, fear, and surprise) or a broader range of emotional expressions at different intensity levels (i.e., 20%, 60%) could extend the current findings in future studies. The fifth limitation is evident from the contradictory results presented in the scientific literature regarding the potential effects of the demographic characteristics on the recognition ability of emotional expressions. Even if the accuracy responses here resulted in being not affected by the stimuli gender, future studies should establish the role that age and gender play in expressing emotions across distinct ethnicities.

### 5.2. Applications and Future Directions

In everyday life, when facial expressions are ambiguous or uninformative, the influence of exposure, culture, and context plays an important role in the emotional processing, which may also lead to biases in explicit judgments. This may be of particular significance in situations perceived as dangerous or threatening such as police or juridical processes where an accurate interpretation of facial emotions is most crucial to society. The current findings apply to real-world applications, from social interactions in general to specific patterns of cross-cultural communication [62]. The evidence that face recognition may depend on the relationship between multiple variables (e.g., gender, ethnicity, emotional valence, and intensity) could disclose the complex interplay of bottom-up (e.g., perceptive) and top-down (e.g., cognitive) processes involved the affective face processing of the observer (e.g., with peculiar perceptual abilities and reappraisal), also influenced by the surrounding environment (e.g., contextual information, social role in the group, cultural background of Western or Eastern society, membership in a collectivist versus individualist society) [63,64]. Peculiar perceptual and cognitive abilities in facial discrimination, influential stereotypes or bias, emotional reappraisal, and arousal of the observer may emerge from explicit (e.g., behavioral) and implicit (e.g., physiological, neurophysiological) processes [27]. The definition of indexes able to measure these processes could stimulate multidisciplinary research in defining the neurophysiological correlates of emotional expression and developing even more accurate automatic emotion recognition systems [65].

Future analysis could be conducted by combining the extracted indexes to structure a dimensional face perception model. The model would evaluate if specific characteristics of the perceived face could modulate its categorization through the dimensions. The collected data could be evaluated across the dimensions of valence (the emotional meaning of the face) and arousal (the behavioral and physiological activation felt approaching or avoiding the face). Potential association and dissociation effects between valence and arousal could disclose further insight into explicit and implicit facial emotional processing.

## 6. Conclusions

Scholars have argued that the production and comprehension of facial emotions may be universal across members of the same species. However, recent studies reported that implicit and explicit processing of emotions is influenced by multiple factors which characterize the facial expressions, such as gender, culture, emotional valence and intensity, all features controlled within this study.

In the current research, male and female participants did not differ in their behavioral performance. Moreover, the accuracy levels were not affected by the gender or the facial expressions’ emotional valence. Contrary to our hypothesis and the pieces of evidence pointing to an own-race bias, we observed that Caucasian emotional faces were judged more accurately than Japanese ones within the Japanese sample. This effect was particularly evident for the behavioral response to ambiguous angry expressions with differences between the two cultures. In line with the hypotheses, ambiguous Caucasian faces also elicited a higher arousal than Japanese ones at a neurophysiological level. Within an ecological perspective, an explanation could be attributed to the potential impact of contextual information (i.e., ethnicity, familiarity) [66] and unpredictable environment (i.e., emotional intensity) [67] on the cognitive states (i.e., reappraisal) and emotional responses (i.e., stress, anxiety) involved in the encoding of facial expressions [68]. At a neural level, this cognitive process may be related to cortical and subcortical cerebral regions, which act as salience [69] and ambiguity detectors [70]. As observed from the unexpected patterns of the Frontal Asymmetry Index, Japanese male participants also exhibited an avoidant response towards angry faces of the same sex and an approaching response towards male happy faces and female angry faces. Males could be evolutionarily predisposed to interpret the female emotional face as less stressful and less hostile than male ones because of the innate predisposition for mating [71].

Overall, our data draw attention to the potential influence of ethnicity and emotional intensity on the implicit and explicit facial expressions’ emotional processing. In conclusion, future research recommendations include larger sample size and data collection from different ethnic groups to investigate the cross-cultural influences on emotional faces expressed with different intensity levels more widely.

## Figures and Tables

**Table 1:** For each generated behavioral variable, the total accuracy mean rate, the male participants’ accuracy mean rate, and the female participants’ accuracy mean rate are reported. For each accuracy mean value, the related standard error mean is indicated between parentheses.

Behavioral Variables	Total	Males	Females
All Faces	0.80 (0.01)	0.80 (0.02)	0.80 (0.02)
Male Faces	0.81 (0.01)	0.80 (0.02)	0.81 (0.02)
Female Faces	0.79 (0.01)	0.80 (0.02)	0.79 (0.02)
Japanese Neutral Faces	0.88 (0.03)	0.92 (0.04)	0.84 (0.05)
Caucasian Neutral Faces	0.94 (0.02)	0.96 (0.02)	0.93 (0.04)
Happy Faces	0.75 (0.03)	0.75 (0.04)	0.75 (0.04)
Angry Faces	0.74 (0.02)	0.71 (0.02)	0.76 (0.02)
Japanese Emotional Faces	0.65 (0.03)	0.61 (0.03)	0.69 (0.03)
Caucasian Emotional Faces	0.84 (0.02)	0.85 (0.02)	0.83 (0.02)
Low Intensity Faces	0.51 (0.03)	0.50 (0.05)	0.53 (0.04)
High Intensity Faces	0.98 (0.01)	0.96 (0.02)	0.99 (0.01)
Japanese Low Intensity Happy Faces	0.53 (0.07)	0.44 (0.10)	0.61 (0.09)
Caucasian Low Intensity Happy Faces	0.50 (0.06)	0.60 (0.07)	0.41 (0.08)
Japanese Low Intensity Angry Faces	0.16 (0.05)	0.12 (0.06)	0.20 (0.07)
Caucasian Low Intensity Angry Faces	0.86 (0.03)	0.83 (0.05)	0.89 (0.03)
Japanese High Intensity Happy Faces	0.99 (0.01)	0.98 (0.02)	1.00 (0.00)
Caucasian High Intensity Happy Faces	0.98 (0.01)	0.96 (0.03)	1.00 (0.00)
Japanese High Intensity Angry Faces	0.94 (0.03)	0.90 (0.05)	0.96 (0.02)
Caucasian High Intensity Angry Faces	1.00 (0.00)	1.00 (0.00)	1.00 (0.00)

## Data Availability

The data and codes of this study can be found in the NTU’s Data repository (DR-NTU Data) at the following address: https://doi.org/10.21979/N9/GTRLJJ (accessed on 20 April 2021).
